# Barrier's deficiency impact in food allergy course in children

**DOI:** 10.1186/2045-7022-5-S1-O17

**Published:** 2015-03-11

**Authors:** Olga Pakholchuk

**Affiliations:** 1 Zaporizhia State Medical University, faculty pediatria department, Zaporizhia, Ukraine

## Background

It is believed that in many cases Food allergy (FA) develops due to the contact of the immune system with different allergens through affected skin and mucous barriers. Studies of the filaggrin mutations in atopic dermatitis showed informativity of the transepidermal water lost measurement (TEWL) in assessment of the skin barrier condition. Faecal calprotectin is marker of the inflammation that can be helpful in understand of the GIT mucous condition.

## Aim

To investigate a possible relationship between the TEWL and stool calprotectin level as markers of the barrier's deficiency with severity of the FA and treatment response.

## Methods

80 patients with skin symptoms of food allergy of different severity were under study. TEWL level, faecal calprotectin level were measured to identify the presence of the skin and mucous barrier's deficiency in children and it's change under treatment.

## Results

High TEWL was associated with a more severe disease course and a poor response to treatment with antihistamines than in patients with less TEWL (r=,5, P<.05). Faecal calprotectin was elevated in 35/80 patients (43.7%) and was found to be significantly higher in patients with more severe FA symptoms (P<.05). Not all patients with positive stool marker (45.7% 16/35) noticed unsteady stool in anamnesis. In addition, the most effective influence on the severity of the TEWL had topical treatment with emollients, than oral intake of the antihistamines (OR 13.5, 95% CI: 4.2-14.6; P<.05).

## Conclusion

TEWL and faecal calprotectin level measurement are simple and informative tests for FA course assess. Patients with FA and deficiency in skin and mucous barrier experience a more severe course of the disease and show a poor response to treatment with antihistamines. In this case it was proposed to use TEWL and faecal calprotectin level measurement as additional tests to reveal and predict possible ways of the further Atopic March in children. Topical treatment with emollients is more effective than oral intake of the antihistamines.

